# The Transcription Factor *Myt3* Acts as a Pro-Survival Factor in β-cells

**DOI:** 10.1371/journal.pone.0051501

**Published:** 2012-12-07

**Authors:** Bryan R. Tennant, Ratib Islam, Marabeth M. Kramer, Yulia Merkulova, Roger L. Kiang, Cheryl J. Whiting, Brad G. Hoffman

**Affiliations:** 1 Child and Family Research Institute, British Columbia Children’s Hospital and Sunny Hill Health Centre, Vancouver, British Columbia, Canada; 2 Department of Surgery, University of British Columbia, Vancouver, British Columbia, Canada; University of Bremen, Germany

## Abstract

**Aims/Hypothesis:**

We previously identified the transcription factor *Myt3* as specifically expressed in pancreatic islets. Here, we sought to determine the expression and regulation of *Myt3* in islets and to determine its significance in regulating islet function and survival.

**Methods:**

*Myt3* expression was determined in embryonic pancreas and adult islets by qPCR and immunohistochemistry. ChIP-seq, ChIP-qPCR and luciferase assays were used to evaluate regulation of *Myt3* expression. Suppression of *Myt3* was used to evaluate gene expression, insulin secretion and apoptosis in islets.

**Results:**

We show that *Myt3* is the most abundant MYT family member in adult islets and that it is expressed in all the major endocrine cell types in the pancreas after E18.5. We demonstrate that *Myt3* expression is directly regulated by Foxa2, Pdx1, and Neurod1, which are critical to normal β-cell development and function, and that Ngn3 induces *Myt3* expression through alterations in the *Myt3* promoter chromatin state. Further, we show that *Myt3* expression is sensitive to both glucose and cytokine exposure. Of specific interest, suppressing *Myt3* expression reduces insulin content and increases β-cell apoptosis, at least in part, due to reduced *Pdx1, Mafa, Il-6, Bcl-xl, c-Iap2* and *Igfr1* levels, while over-expression of *Myt3* protects islets from cytokine induced apoptosis.

**Conclusion/Interpretation:**

We have identified *Myt3* as a novel transcriptional regulator with a critical role in β-cell survival. These data are an important step in clarifying the regulatory networks responsible for β-cell survival, and point to *Myt3* as a potential therapeutic target for improving functional β-cell mass.

## Introduction

Our understanding of the transcriptional networks regulating gene expression during β-cell genesis and function is rapidly expanding [1], [2], [3], [4]. The importance of these networks is exemplified by the fact that several monogenic forms of diabetes are linked to defects in transcription factors, namely *Hnf4α* (MODY1), *Hnf1α* (MODY3), *Pdx1* (MODY4), *Hnf1β* (MODY5) and *Neurod1* (MODY6) [5], [6], [7], [8], [9]. In addition, transcription factors play critical roles in glucose-stimulated insulin secretion, via the regulation of vesicle docking (*Foxa2*), glucose sensing (*Pdx1*), glucose-, KCl- and arginine-induced insulin secretion (*Mafa*), oxidative metabolism of glucose, and insulin secretion complex formation (*Neurod1*) [10], [11], [12], [13]. Despite this, our understanding of these processes is far from complete and we anticipated that the identification of novel transcriptional regulators expressed specifically in β-cells, and the determination of their functional roles would help further elucidate these complex networks.

In previous work [2], we identified 2,536 genes with pancreas-enriched expression, including *Myelin transcription factor 3* (*Myt3*), also known as *Suppression of tumourigenicity 18* (*St18*). *Myt3* is part of the C2HC-type zinc-finger, or MYT, family of transcription factors that in vertebrates is composed of three genes: *Myt1, Myt1l* and *Myt3* [14], [15], [16]. These factors function as both positive and negative regulators of gene expression [14], [15], [16], [17]; and both *Myt1* and *Myt1l* have been implicated in the regulation of neuronal cell fate determination, proliferation and differentiation [14], [15], [18], [19]. *Myt3* was initially identified as a transcriptional repressor in rat brain that strongly bound to bipartite AAASTTT motifs [16]. *Myt3* suppression and promoter hypermethylation were subsequently determined to be prevalent in primary breast tumours [20], while *Myt3* degradation by miR-125b-2 was implicated in the development of megakaryoblastic leukaemia [21]. In addition, in dermal fibroblasts *MYT3* regulates TNFα induced pro-inflammatory and pro-apoptotic gene expression, including *Il-1α* and *Il-6* [22].

In pancreas, to date, studies on the MYT family of transcription factors have been limited to *Myt1*. These studies determined that a Myt1/Ngn3 feed forward loop is required for pancreatic endocrine cell specification, and as a result *Myt1* disruption results in impaired endocrine cell function, including glucose tolerance and insulin secretion [23], [24], [25]. Interestingly, *Myt3* expression is up-regulated in endocrine cells lacking *Myt1* [25], suggesting *Myt3* plays a compensatory role. Despite these findings no previous studies have assessed *Myt3’s* significance in pancreatic islet function. To address this deficit we assess *Myt3’s* expression in pancreas development, it’s regulation by key transcription factors, and its role in islet function and survival.

## Methods

### In situ Hybridisation and Immunofluorescence

Probes for *in situ* hybridization were generated using the primers: *Myt3* forward: 5′-ggctgccaaaagacagaaag-3′; reverse: 5′-agttcatggccgtagtgacc-3′ and cloned into pCRII-TOPO (Invitrogen). RNA probes were subsequently labeled with DIG-UTP using T7/SP6 polymerase reactions with 1 µg of linearized plasmid (Roche). *In situ* hybridization of E9.5, E14.5 embryo and isolated islet sections was performed as described in Prado *et al.* [26]. In short, cryostat sections (10 µm) were treated with 1 µg/ml proteinase K and fixed in 4% paraformaldhyde. Sections were hybridized with 1 µg/ml of probe overnight at 70°C. High stringency washes were used to remove unbound probe. Sections were subsequently blocked with 10% FBS, 1% Blocking Reagent (Roche) and incubated with anti-digoxigenin-alkaline phosphatase antibody diluted 1∶1000. Slides were washed and color developed using BM purple as a substrate (Roche).

Immunohistochemistry was performed on islet cryo-sections following *in situ* hybridisation. Sections were stained with guinea pig anti-Insulin (1/100; Stem Cell Technologies Inc.) or guinea pig anti-Glucagon (1/500; Linco). Immunohistochemistry was also performed on paraffin sections of E14.5 mouse embryos, as well as E16.5, E18.5 and adult ICR pancreata. Sections were co-stained with rabbit anti-Myt3 (1/250) and guinea pig anti-Insulin (1/1000; Linco), guinea pig anti-Glucagon (1/1000; Linco), guinea pig anti-PP (1/100; Linco), goat anti-Somatostatin (1/1000; Santa Cruz) or mouse anti-Pdx1 (1/500; DSHB). Primary antibodies were detected using donkey anti-rabbit Alexa 488, goat anti-guinea pig Alexa 546, goat anti-mouse Alexa 546 or donkey anti-goat Alexa 546 (1/2000; Invitrogen). The Myt3 antibody was generated by OpenBiosystems and was raised against the synthetic peptide RKGGIKMTPTKEEKEDSELR. The serum from the terminal bleed of two rabbits was affinity purified.

### Mouse Maintenance, Islet Isolations and Cell Culture

Mice were maintained according to the guidelines of the Canadian Council on Animal Care. All protocols were approved by the UBC Animal Care Committee. Hand-picked pancreatic islets were isolated as previously described [27] and cultured in RPMI 1640 (2g/L Glucose) supplemented with 10% FBS, 50U/ml Penicillin/Streptomycin and 2 mM L-Glutamine at 37° in a 5% CO_2_ humidified incubator. mPAC cells were maintained in Dulbecco’s Modified Eagle Medium (DMEM, 4.5 g/L Glucose) supplemented with 10% FBS, 50 U/ml Penicillin/Streptomycin and 2 mM L-Glutamine (DMEM Complete) at 37° in 5% CO_2_ humidified incubator. Islets were cultured in 3 mM, 7 mM, 11 mM, 16.7 mM and 33 mM glucose, or with various cytokine combinations (INFγ (1000U/ml), IL-1β (17.5 ng/ml) and TNFα (10 ng/ml)) as appropriate. For cycloheximide (CHX) experiments, islets were preincubated in 3 mM glucose for 6 hrs and CHX (10 µg/ml) or DMSO was added 1 hr prior to transferring islets to fresh 3 mM or 16.7 mM glucose supplemented with CHX or DMSO.

### Database Analysis (SAGE and ChIP-seq)

Serial Analysis of Gene Expression (SAGE) data were obtained from the Mouse Atlas of Gene Expression Database (www.mouseatlas.org) [2]. Foxa2 and Pdx1 Chromatin Immunoprecipitation (ChIP) sequencing data were obtained from the Short Read Archive (SRX003306 and SRX003296) [28]. Mafa and Neurod1 ChIP sequencing data were obtained from the Gene Expression Omnibus (GSE30298). Data were analyzed as previously described [2], [28].

### Adenoviral Mediated Knockdown and Over-expression

pLKO.1 vectors containing short hairpin constructs targeting *Myt3* under control of the hU6 promoter and a scramble shRNA construct were purchased from OpenBiosystems. U6-shRNA expression cassettes for three of these were cloned into pAdTrack using InFusion cloning (Clontech) and sequence verified (TRCN0000042478: CCGGCGCAACACTCACAGAAGTCTTCTCGAGAAGACTTCTGTGAGTGTTGCGTTTTTG, TRCN0000042479: CCGGGCAGCAGTATCCAGTCTTTAACTCGAGTTAAAGACTGGATACTGCTGCTTTTTG, TRCN0000042481: CCGGCGAATCCACGACAAGTCTATACTCGAGTATAGACTTGTCGTGGATTCGTTTTTG). pAd-Track-hU6-shRNAs were linearized with PmeI and inserted into the pAd-Easy viral genome by homologous recombination to generate pAdV-sh*Myt3* and pAdV-sh*Scramble* [29]. Full length *Myt3* was cloned into pcDNA3.1-V5/His6 (Invitrogen) and pAdV-Myt3 was generated as above. Islets were transduced with these adenoviruses at the indicated MOI’s for 3 hours and analyzed 48 hrs later. For mPAC studies, cells were plated at 40000 cells/well and transduced with pAdV-*Ngn3* or pAdV-*βgal* for 3 hrs at an MOI of 50 and analysed 48 hrs later [30].

### ChIP-qPCR

Islets were used in ChIP reactions as previously described [28], with 3 µg of anti-Foxa2 (Santa Cruz, sc-6554), anti-Pdx1 (Upstate, 07-696), or anti-Neurod1 (Cell Signalling, D35G2). mPAC cells transduced with pAdV-*Ngn3* or pAdV-*βgal*, as described above, were used in ChIP reactions with 3 µg anti-H3K4me1 (Abcam, Ab8895), anti-H3K4me3 (Abcam, Ab8580), anti-H3K27ac (Abcam, Ab4729), anti-H3K27me3 (Abcam, Ab6002) or rabbit IgG (Santa Cruz, sc-2027). Fold enrichment was calculated relative to the IgG ChIP and percent recovery was calculated relative to sample input.

### Reporter Constructs

A 1200 bp region upstream of the *Myt3* transcriptional start site (TSS) was amplified from mouse genomic DNA and cloned into pGL3-Basic (Promega) to generate the *Myt3* reporter construct. The Foxa2, Pdx1 and Neurod1 binding site mutagenesis primers were designed using the Agilent QuikChange Primer Design tool. Site directed mutagenesis PCR was performed using Phusion Taq (Finnzymes).

### Luciferase Assays

mPAC cells were transfected with 400 ng of pGL3-*Myt3*-promoter dual luciferase reporter construct, pGL3-*Myt3*-promoter mutant constructs or a control pGL3-Basic vector, with or without 200 ng of *Foxa2, Pdx1* or *Neurod1*. An *EGFP* vector was used to ensure equal amounts of DNA were transfected into each well. After 48 hrs reporter activity was analyzed using the Promega Dual Luciferase kit as per manufacturer’s instructions using a Spectramax L luminometer (Molecular Devices).

### qPCR Analysis

Islets were transduced with pAdV-sh*Myt3* clone 2 and pAdV-sh*Scramble* as above. After 48 hrs islets were dispersed and sorted to obtain EGFP positive cells (i.e. transduced cells) using a FACSVantage (BD Biosciences). RNA was isolated from pancreata of E11.5, E13.5, E15.5 and E18.5 embryos as well as ducts and adult islets using Trizol (Invitrogen) and the Qiagen RNA purification kit. mPAC cells were transduced with pAdV-*Ngn3* and pAdV-*βgal* and 48 hrs later were trypsinized and RNA isolated using Trizol (Invitrogen) and the Qiagen RNA purification kit. cDNA was generated using Superscript III (Invitrogen). Taqman probes were used to quantify *Myt3, Myt1, Ins1*, *Ins2*, *Pdx1*, *Neurog3, Pax4, NeuroD1, Il-1β, Il-1α, Il-1rn, Il-6, Tnf-α, iNOS, β-actin,* and *Gapdh* (Applied Biosystems), all other primers were designed using Primer3plus. A Viia7 real-time PCR system (Applied Biosystems) and SYBR Green supermix or Universal PCR Master Mix (Applied Biosystems) was used for all reactions. 10 ng of cDNA was used in each reaction with all reactions done in triplicate. *β-actin* or *Gapdh* were used as internal controls and the change in expression was calculated using 2^−ΔΔCt^.

### Western Blot Analysis

Cell lysates were prepared from islets by sonication in RIPA buffer (Thermo Scientific). 25 µg of total protein was loaded in each well of a 4–12% NuPAGE Bis-Tris gel (Invitrogen). Membranes were probed with antibodies against: Myt3 (1/2000; Open Biosytems), Mafa (1/400; Abcam), and Pdx1 (1/500; Upstate). Blots were subsequently stripped and re-probed with anti- β-actin (1/500; Santa Cruz). Donkey anti-Rabbit (Santa Cruz) and Rabbit anti-Goat (Santa Cruz) secondary antibodies were used at 1/10000.

### Insulin Secretion Assay

Fifty islets per well in a 24 well plate were transduced as above and were subsequently washed and equilibrated in Kreb’s Ringer Buffer (KRB) (115 mM NaCl, 5 mM KCl, 24 mM NaHCO3, 2.5 mM CaCl2, 1 mM MgCl2, 10 mM HEPES and 2% w/v BSA) with 2.8 mM glucose for 1 hr before being transferred into 500 µL KRB with either 2.8 mM glucose, 16.7 mM glucose, 30 mM KCl or 10 mM arginine for a further 1 hr. Supernatants were collected to measure insulin secretion and islets were lysed in 50 µL RIPA buffer with 1× Halt protease inhibitor cocktail (Thermo Scientific) to measure cellular insulin. All samples were analysed using the Insulin (Mouse) ELISA (Alpco) and plates were read using a Spectramax 190 plate reader (Molecular Devices).

### Statistical Analysis

For ChIP-qPCR p-values for enrichment over a negative control region were calculated using a Kruskal-Wallis test with a Dunn’s multiple comparison on 2^−ΔCt^ values, data are presented as fold-enrichment over a negative region +/− SD. For luciferase data relative luciferase activity values were compared using unpaired, two-tailed Student’s t-tests, data are represented as mean +/− SD. For qPCR experiments paired or unpaired, two-tailed Student’s t-tests were used to compare ΔC_T_ values as appropriate. Data are presented as relative quantification values with upper and lower limits. Relative density values for western blot bands were analysed using paired, two-tailed Student’s t-tests and data are represented as mean +/− SEM. P-values for TUNEL positive cells were calculated using paired, two-tailed Student’s t-tests on percent TUNEL positive values. Data are represented as mean +/− SEM. In all cases * indicates a statistically significant difference at p≤0.05, ** at p≤0.01, *** at p≤0.001.

## Results

### *Myt3* is the Dominant MYT Family Member in Mature Islets

**Figure 1:**
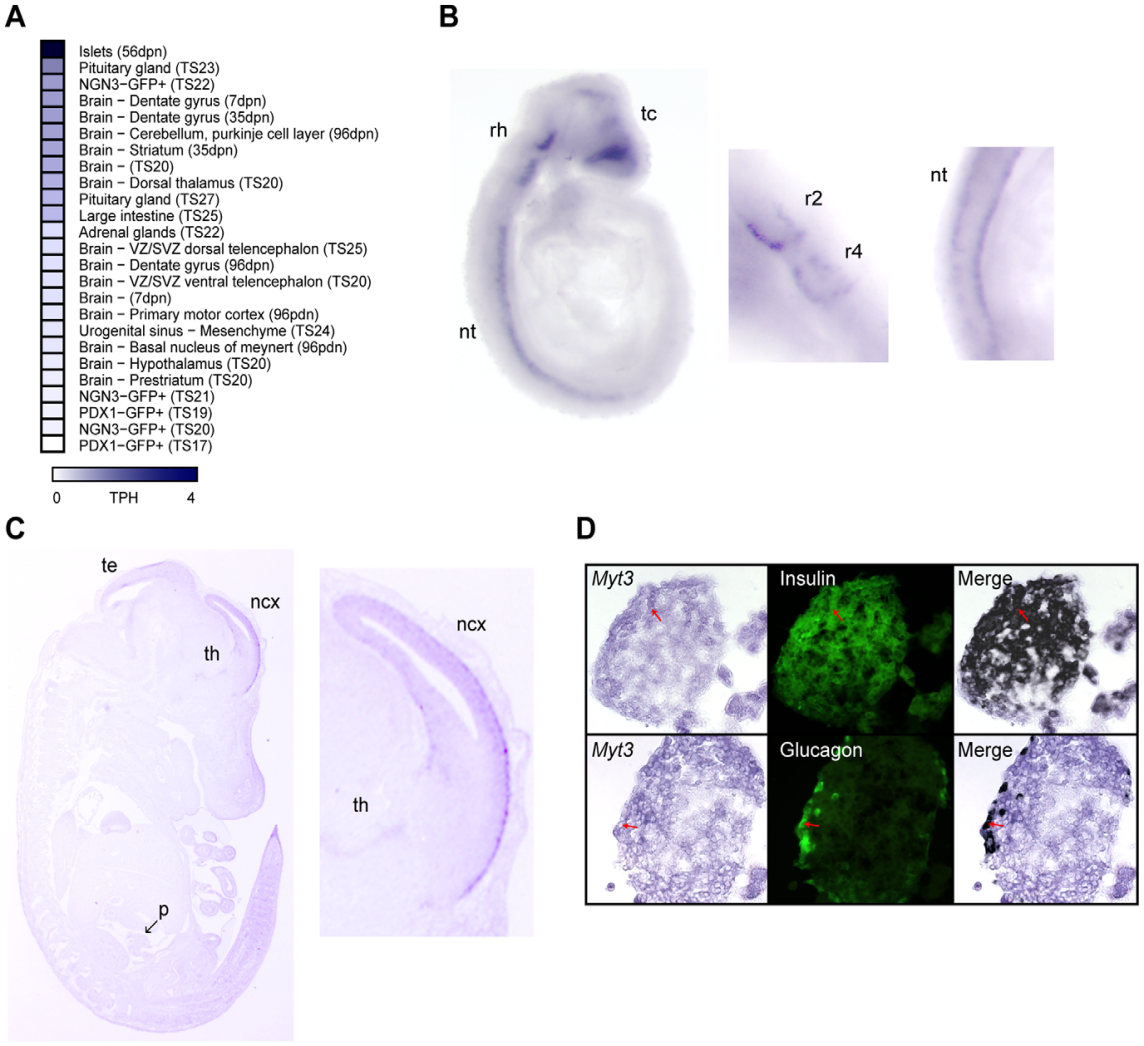
Developmental expression of *Myt3* is restricted to specific cell types. **A)** A heatmap showing the relative gene expression of *Myt3* in different tissues as determined by SAGE analysis of 205 Mouse Atlas of Gene Expression libraries. Tissues with no detected expression are not shown. *Myt3* expression was determined by *in situ* hybridisation of saggital sections of **B)** E9.5 and **C)** E14.5 mouse embryos. **B)** At E9.5 *Myt3* expression is restricted to the neural tube, second and fourth rhombomere and the telencephalon. **C)** *Myt3* expression is evident in the tectum, thalamus and neocortex in E14.5 embryos. *Myt3* is absent from the pancreas (arrow) at this time point. **D)** Combination of immunohistochemical analysis and *in situ* hybridisation demonstrates expression of *Myt3* in insulin and glucagon expressing cells. Co-expression in the merged image is indicated by black pseudo-colouring.

In previous studies *Myt3* was reported to be absent from the developing pancreas [25], although our data suggested its enriched expression in mature pancreatic islets [2]. To confirm our previous data, and clarify the expression of *Myt3* in the pancreas, we assessed its expression in 205 serial analysis of gene expression (SAGE) libraries. We found *Myt3* SAGE tags (representing *Myt3* expression) in neural tissue, as well as at low levels in pancreatic and endocrine precursor cells. However, in confirmation of our previous results, maximal *Myt3* levels were found in pancreatic islets (Figure 1A). To further validate these data, we performed *in situ* hybridisation on mouse embryo’s at embryonic day 9.5 (E9.5) and 14.5 (E14.5), as well as on adult islets (Figure 1B–D). Whole mount *in situ* hybridization with E9.5 embryo’s showed strong *Myt3* staining in the telencephalon, the second and fourth rhombomeres, as well as in the ventral neural tube (Figure 1B). At E14.5 we found relatively strong *Myt3* staining in the anterior of the neocortex, with weaker staining in the thalamus and tectum (Figure 1C). In agreement with previous studies [25], no staining was found in the pancreas at this time point. Despite this, we found strong *Myt3* staining in mature pancreatic islets, which co-localized with both insulin and glucagon (Figure 1D). These data demonstrate that although *Myt3* expression is minimal in the developing pancreas it is relatively abundant in mature islets.

**Figure 2:**
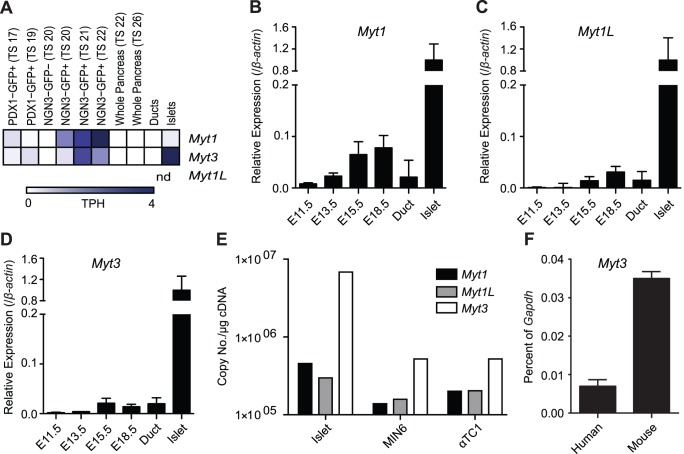
*Myt3* is the dominant MYT family member in adult islets. **A)** A heatmap showing the relative gene expression of *Myt1*, *Myt1L* and *Myt3* in pancreatic and endocrine precursor cells, whole pancreas, duct cells and mature islets as determined by analysis of 10 SAGE libraries (www.mouseatlas.org). Expression levels for **B)** *Myt1*, **C)** *Myt1L* and **D)** *Myt3* in the pancreas as determined by qPCR at various stages of embryonic development as wells as in ductal cells and whole islets from adult mice (8–10 weeks of age). Expression is relative to *β-actin* and is normalised to expression levels in whole islets. **E)** Absolute level of *Myt1, Myt1L* and *Myt3* transcripts in islets, MIN6 cells (a β-cell line) and αTc1 cells (an α-cell line) as determined by qPCR. Absolute quantification expressed as number of copies per µg cDNA. **F)** Expression of *Myt3* in human and mouse islets as a percentage of *Gapdh* expression.

Given the high degree of similarity between the MYT family members [16], and their possible functional redundancy [16], [31], we wanted to determine which family member was most abundant in developing pancreas tissues and in adult islets. Using our SAGE data we determined that while *Myt1* is more highly expressed in *Ngn3* expressing endocrine precursor cells, *Myt3* is more abundant in mature islets (Figure 2A). *Myt1l* could not be assessed as it does not produce any SAGE tags that uniquely map to it. In agreement, qPCR analysis of *Myt1*, *Myt1l* and *Myt3* in developing pancreas tissues and adult islets showed that *Myt1* was clearly more abundant than *Myt1l* or *Myt3* in the developing pancreas, particularly at E15.5 and E18.5 (Figure 2B–D). However, all three family members showed maximal expression in adult islets (Figure 2B–D), likely due to the higher proportion of cells expressing these factors in islets as compared to within the whole developing pancreas. To clarify which of the MYT family members is dominant in adult islet cells, we determined the total copy number of *Myt1*, *Myt1l*, and *Myt3* transcripts in islets, as well as in MIN6 (β-cell) and αTC1 (α-cell) cells using absolute quantification qPCR. *Myt3* was expressed at a 15-fold higher level in islets, a 4-fold higher level in MIN6 cells, and a 2.5-fold higher level in αTC1 cells than *Myt1*, and a 23-fold higher level in islets, a 3-fold higher level in MIN6 cells, and a 2.5-fold higher level in αTC1 cells than *Myt1l* (Figure 2E). Last, we sought to determine the expression level of *Myt3* in human islets relative to mouse islets, and found that although *Myt3* is expressed in human islets, *Myt3* expression is 4-fold higher in mouse islets (Figure 2F). Together, these data demonstrate that *Myt3* is more abundantly expressed in mature pancreas endocrine cell types than either *Myt1* or *Myt1l*, and confirm its expression in α- and β-cells.

### Myt3 is Expressed in Maturing and Adult Endocrine Cells

**Figure 3:**
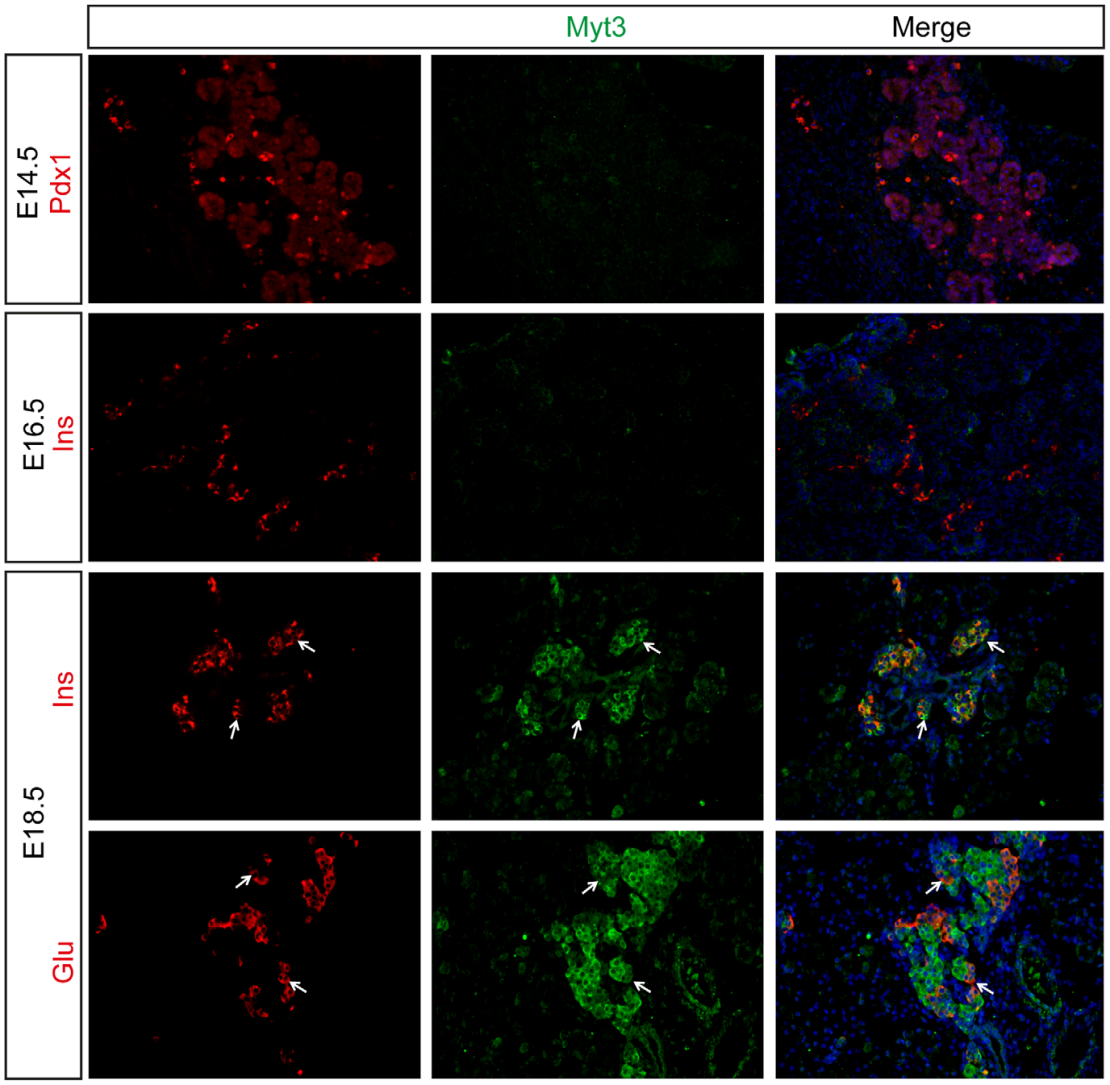
Myt3 protein is detected in endocrine cells from E18.5. Saggital sections of E14.5, E16.5 and E18.5 pancreata were analysed for expression of Insulin, Glucagon or Pdx1, as indicated (red), and Myt3 (green). Nuclei were stained with Hoechst (blue). Arrows indicate co-localisation of Myt3 with indicated endocrine markers.

**Figure 4:**
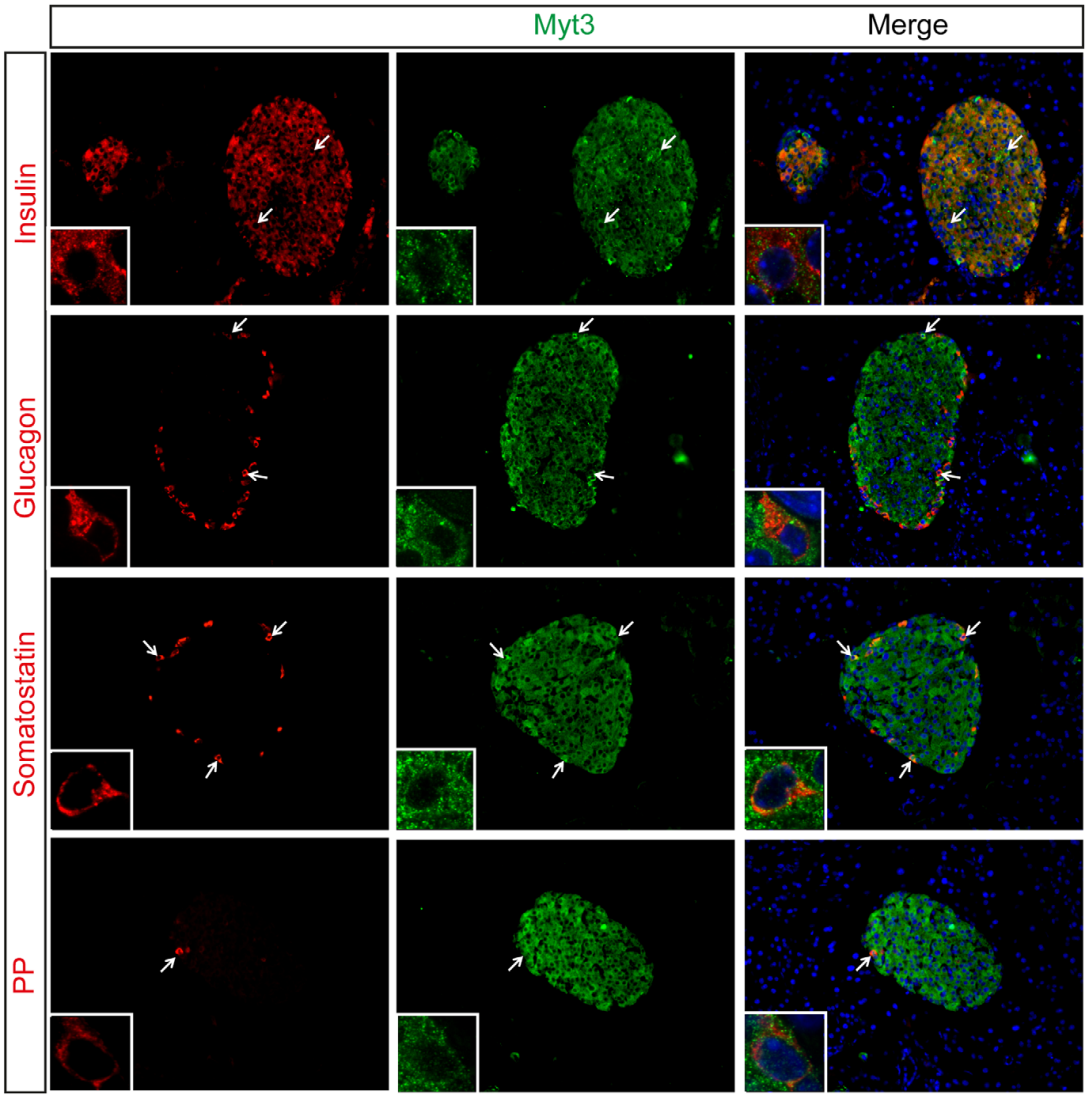
*Myt3* co-localises with endocrine cell markers in adult pancreas. Saggital sections of adult pancreata were analysed for expression of Insulin, Glucagon, Somatostatin and Pancreatic Polypeptide, as indicated (red), and Myt3 (green). Nuclei were stained with Hoechst (blue). Arrows indicate co-localisation of Myt3 with indicated endocrine markers. High magnification confocal images of individual cells showing co-localization of Myt3 with Insulin, Glucagon, Somatostatin and Pancreatic Polypeptide, representative cells are shown (Inset), note that Myt3 staining is predominately cytoplasmic but can also be found within the nucleus.

The above data indicate that *Myt3* expression occurs predominantly in adult islet cell types. To determine whether Myt3 protein levels match this pattern, and to identify the cell types that Myt3 is expressed in, we developed an antibody against it. Using this antibody we found no evidence of Myt3 protein in the developing pancreas at either E14.5 or E16.5 (Figure 3). At E18.5 however, Myt3 protein was found in both insulin (β-cells) and glucagon (α-cells) expressing cells (Figure 3). Similarly, Myt3 staining was evident throughout the islet in adult pancreas sections, while no Myt3 staining was evident in the surrounding exocrine tissue (Figure 4). Similar to what we observed in our *in situ* experiments with whole islets, co-staining of adult sections with endocrine cell markers showed that Myt3 co-localizes in cells expressing insulin (β-cells), glucagon (α-cells), somatostatin (δ-cells) and pancreatic polypeptide (PP-cells) (Figure 4). High magnification confocal microscopy confirmed the co-localization of Myt3 with endocrine markers, and indicated that in mature endocrine cell types Myt3 is primarily cytoplasmic, with only a fraction of total protein localizing to the nucleus (inset), similar to other β-cell transcription factors such as Pdx1 and Neurod1 [32], [33]. These data indicate that Myt3 is first evident at E18.5, and that it is expressed in mature α-, β-, δ-, and PP-cell types.

### *Myt3* Expression is Regulated by Foxa2, Pdx1 and Neurod1

**Figure 5:**
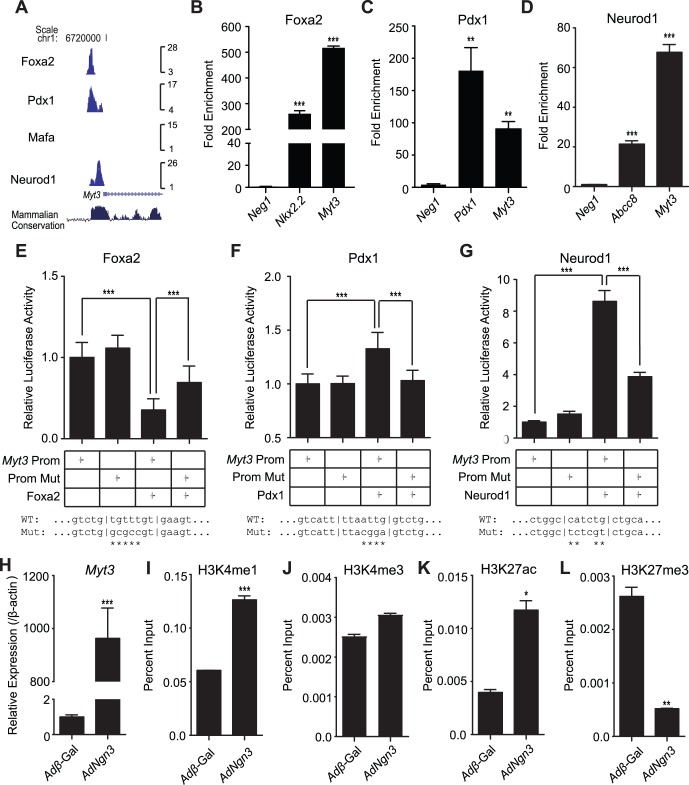
*Myt3* expression in islets is under the control of known regulators of β-cell function. **A)** A screenshot of the *Myt3* promoter region in the UCSC genome browser showing Foxa2, Pdx1, Mafa and Neurod1 ChIP-seq data from islets. Peaks indicate binding sites. ChIP-qPCR was used to validate **B)** Foxa2, **C)** Pdx1 and **D)** Neurod1 binding within the *Myt3* promoter region. *Nkx2.2*, *Pdx1* and *Abcc8* are positive controls for Foxa2, Pdx1 and Neurod1 binding respectively. **E–G)** Relative luciferase activity of the indicated luciferase reporter vectors co-transfected with empty vector or with Foxa2, Pdx1 or Neurod1 expressing vectors. Mutant vectors had the indicated transcription factor binding sites altered by site-directed mutagenesis. Wild type and mutant binding site sequences are as indicated. **H)** *Myt3* expression relative to *β-actin* following treatment of mPAC cells with pAdV-*Ngn3*. ChIP-qPCR was used to determine **I)** H3K4me1, **J)** H3K4me3, **K)** H3K27ac and **L)** H3K27me3 histone modifications at the *Myt3* promoter. * indicates a statistically significant difference at p≤0.05, ** at p≤0.01, and *** at p≤0.001 based on student’s t-test for luciferase data and a Kruskal-Wallis test with a Dunn’s multiple comparison for ChIP-qPCR data.

To characterise the factors responsible for the regulation of *Myt3* expression we first assessed Foxa2, Pdx1, Neurod1 and Mafa ChIP-seq data generated from islets [28]. We identified Foxa2, Pdx1 and Neurod1 enrichment, or peaks, in the *Myt3* promoter region (Figure 5A) suggesting its expression is directly regulated by these factors. No enrichment of Mafa was noted. To validate these data we used ChIP-qPCR. Using an antibody against Foxa2 we obtained a 250-fold (p≤0.01, n = 3) enrichment of an *Nkx2.2* positive control region [2], and a 500-fold (p≤0.01, n = 3) enrichment of the *Myt3* promoter (Figure 5B). Meanwhile, using an antibody against Pdx1 we obtained a 180-fold (p≤0.01, n = 3) enrichment in a *Pdx1* positive control region [2], and a 90-fold (p≤0.01, n = 3) enrichment of the *Myt3* promoter (Figure 5C); and using an antibody against Neurod1 we obtained a 21-fold (p≤0.001, n = 3) enrichment of an *Abcc8* control region, and a 70-fold (p≤0.001, n = 3) enrichment of the *Myt3* promoter (Figure 5D). In all cases less than a 5-fold enrichment was obtained using primers for regions upstream of the *Myt3* promoter. To further confirm the direct regulation of *Myt3* expression by these factors we generated a *Myt3*-promoter luciferase reporter. In co-transfections with this reporter, Foxa2 reduced *Myt3* promoter activity by 1.3-fold (p≤0.001, n = 3), while Pdx1 and Neurod1 increased promoter activity by 1.3-fold (p≤0.001, n = 3) and 9-fold (p≤0.001, n = 3), respectively (Figure 5E–G). Mutation of the Foxa2 binding site reversed the suppressive effect of Foxa2 by 2-fold (p≤0.001, n = 3), while mutation of the Pdx1 and Neurod1 binding sites reduced the relative luciferase activity by 3-fold (p≤0.001, n = 3) and 3.4-fold (p≤0.001, n = 3), respectively, over the non-mutated promoter (Figure 5E–G). Together, these data show that Foxa2, Pdx1 and Neurod1 directly regulate *Myt3* expression, and that Neurod1 is likely a primary determinant of *Myt3* promoter activity.

Genes regulated by Neurod1 in mature tissues are often initially induced during development by the related bHLH transcription factor Ngn3, which is critical to pancreas endocrine cell specification [34], as both bind to E-box elements [35], [36]. Thus, to test whether *Ngn3* induces *Myt3*, we treated mPAC cells with an *Ngn3* over-expressing adenovirus, or control *βgal* expressing virus. *Ngn3* over-expression resulted in a 963-fold (p≤0.0001, n = 4) increase in *Myt3* expression relative to cells treated with the *βgal* virus (Figure 5H). We next evaluated the ability of *Ngn3* over-expression to alter the histone modification status of the *Myt3* promoter to establish the mechanism of *Myt3* induction. We performed ChIP-qPCR for mono-methylated Histone 3 Lysine 4 (H3K4me1) (Figure 5I), tri-methylated Histone 3 Lysine 4 (H3K4me3) (Figure 5J) and acetylated Histone 3 Lysine 27 (H3K27ac) (Figure 5K), which demarcate active cis-regulatory loci [37], [38], [39], [40]; as well as, for tri-methylated Histone 3 Lysine 27 (H3K27me3) (Figure 5L), which is associated with repressed chromatin [41], [42], [43]. Our data demonstrate *Ngn3* over-expression in mPAC cells increased the levels of H3K4me1 and H3K27ac by 2-fold (p≤0.0001, n = 3) and 3-fold (p≤0.05, n = 3) respectively. Meanwhile levels of tri-methylated Histone 3 Lysine 27 (H3K27me3) were reduced 5.0-fold (p≤0.01, n = 3) relative to *βgal* expressing cells. Levels of tri-methylated Histone 3 Lysine 4 (H3K4me3) were unchanged. These data suggest that *Ngn3* expression alters the epigenetic landscape around the *Myt3* promoter from an inactive, to an active chromatin state, thereby initiating its expression.

### *Myt3* Expression is Regulated by Glucose and Cytokines

**Figure 6:**
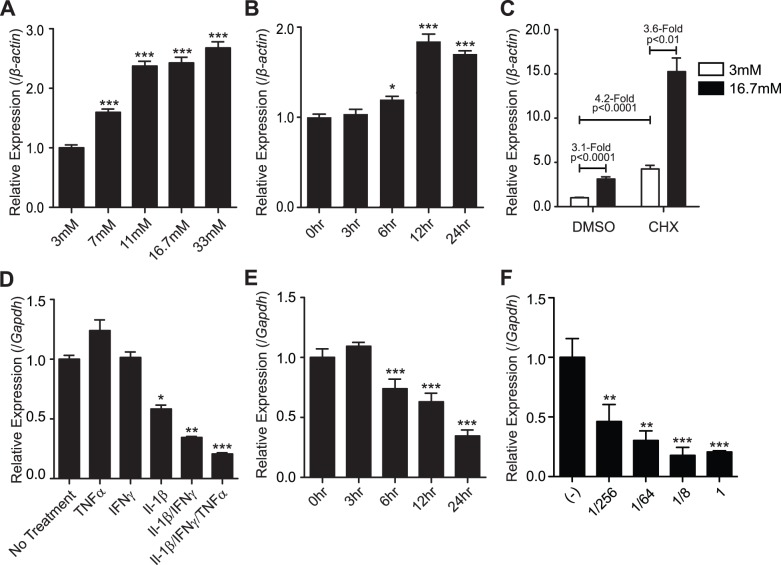
*Myt3* expression is sensitive to both glucose and cytokine exposure. **A)** Whole cultured islets were treated with the indicated glucose concentrations for 24 hrs, after being pre-incubated for 24 hr in 3 mM glucose. Subsequently qPCR was used to determine the relative expression of *Myt3* as compared to *β-actin*. **B)** *Myt3* expression was determined at the indicated time points following transfer into 16.7 mM glucose. **C)** *Myt3* expression was determined for islets incubated in low or high glucose following treatment with DMSO or cycloheximide (10 µg/ml). Expression is expressed relative to *β-actin*. **D)** Whole cultured islets were treated with the indicated cytokine combinations, or **E)** with triple cytokine mix at the indicated time points. *Myt3* expression is expressed relative to *Gapdh*. **F)** Whole cultured islets were treated with the indicated cytokine doses (a dose of “1” equals 1000U/ml INFγ, 17.5 ng/ml IL-1β and 10 ng/ml of TNFα) for 24 hours. Subsequently qPCR was used to determine the relative expression of *Myt3*. Expression is expressed relative to *Gapdh*. * indicates a statistically significant difference at p≤0.05, ** at p≤0.01, and *** at p≤0.001, based on student’s t-test.

Under normal physiological conditions islets are exposed to fluctuating concentrations of glucose and many genes with critical roles in controlling islet function, such as *Insulin*, *Iapp* and *Mafa*, are regulated by glucose [32], [44], [45], [46]. To determine whether *Myt3* is similarly regulated we assessed its expression in islets at various glucose concentrations 24 hrs after transfer from 3 mM glucose. Exposure of islets to 7 mM, 11 mM, 16.7 mM and 33 mM glucose increased *Myt3* expression by 1.78- (p≤0.001, n = 4), 2.74- (p≤0.001, n = 4), 2.71- (p≤0.001, n = 4) and 2.86-fold (p≤0.001, n = 4), respectively, over 3 mM glucose (Figure 6A). We next sought to determine the timing of the increase in *Myt3* expression in response to glucose. 3 hr after transfer to 16.7 mM glucose there was no change in *Myt3* expression, and only a slight but significant (1.2-fold, p≤0.05, n = 4) change by 6 hrs; however, by 12 hrs *Myt3* had reached maximal induction (1.84-fold, p≤0.001, n = 4) and this was maintained at 24 hrs (1.70-fold, p≤0.001, n = 4) (Figure 6B). The delay in glucose induced *Myt3* expression suggests that it may be dependent on the synthesis of additional regulatory proteins in addition to the translocation of transcription factors to the nucleus. To test this we treated islets with cycloheximide (CHX, 10 µg/ml) to inhibit protein synthesis. Interestingly, treatment with CHX increased basal *Myt3* expression by 4.2-fold (p≤0.001, n = 3) relative to 3 mM glucose with DMSO. Induction with 16.7 mM glucose increased *Myt3* levels a further 3.6-fold (p≤0.01, n = 3), similar to the level of *Myt3* induction by 16.7 mM glucose in DMSO (3.2 fold, p≤0.001, n = 3) (Figure 6C). These data indicate that *Myt3* expression is positively regulated by the glucose signals responsible for insulin secretion, and suggest that *Myt3* is repressed by some factor that requires continued protein synthesis.

In both type 1 and type 2 diabetes β-cell exposure to cytokines can induce dysfunction by altering the expression of genes responsible for regulating normal β-cell function [47], [48]. In fibroblasts *Myt3* was found to be up-regulated by exposure to TNFα [22], but to be down-regulated in a microarray study of genes affected by exposure to Il-1β and IFNγ in rat islets [48]. To clarify this discrepancy, we examined the expression level of *Myt3* following exposure of islets to different combinations of Il-1β, IFNγ, and TNFα. *Myt3* expression was reduced by exposure of islets to IL-1β (1.7 fold, p≤0.05, n = 3) but not by IFNγ or TNFα, while a combination of Il-1β and IFNγ reduced *Myt3* expression 3-fold (p≤0.01, n = 3). Treatment of islets with Il-1β, IFNγ and TNFα together had the most significant effect, reducing *Myt3* expression 5-fold (p≤0.001, n = 3) (Figure 6D). Similar to what was seen following exposure of islets to glucose, the reduction in *Myt3* expression was also time dependent. At 3 hrs post transfer into a full dose of cytokine mix *Myt3* expression was unchanged. By 6 hrs post transfer *Myt3* expression was significantly reduced (1.35-fold, p≤0.001, n = 3) with maximal suppression being reached by 24 hrs (2.9-fold, p≤0.001, n = 3) (Figure 6E). To determine how *Myt3* expression varied with cytokine dose dependent we treated islets with varying concentrations of the triple cytokine mix. Our data demonstrate that maximal reduction in *Myt3* levels was evident at 1/8 the concentration of Il-1β, IFNγ and TNFα used above (i.e 125 U/ml INFγ, 2.15 ng/ml IL-1β and 1.25 ng/ml TNFα) (Figure 6F).

**Figure 7:**
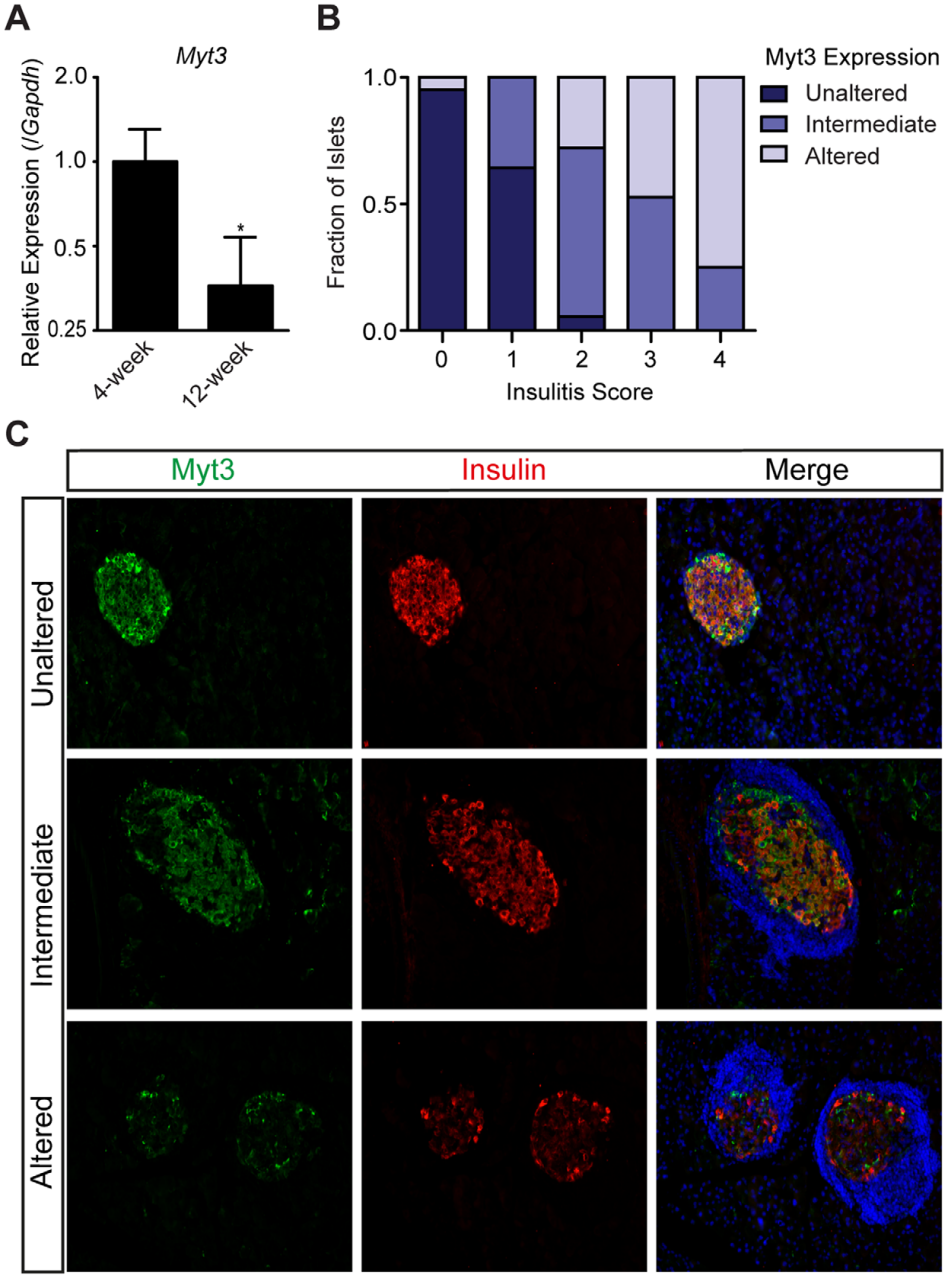
Exposure of islets to cytokines in a mouse model of T1D decreases *Myt3* expression. **A)** RNA was isolated from whole pancreas of female 4-week and 12-week old NOD mice and *Myt3* expression was determined relative to *Gapdh*. **B)** Insulitis levels and Myt3 expression in islets were scored by analysing sections from 12-week old NOD mice. **C)** Representative images of NOD sections showing Myt3 (green) and Insulin (red) expression in islets that are unaltered, intermediately altered and altered. Nuclei were stained with Hoechst (blue). * indicates a statistically significant difference at p≤0.05 based on student’s t-test.

As Il-1β, IFNγ and TNFα are important cytokine effectors of β-cell death in type 1 diabetes [47], [48], we next sought to determine whether *Myt3* is reduced by immune-cell attack in non-obese diabetic (NOD) mice. We isolated RNA from whole pancreata from 4-week old pre-diabetic and 12-week old diabetic female NOD mice and analysed *Myt3* expression. Our data demonstrate that in pancreata from diabetic mice undergoing immune infiltration *Myt3* expression is reduced by 2.5-fold (p≤0.05, n = 4) (Figure 7A). We also assessed Myt3 expression relative to the level of immune infiltration by immunofluorescence. For this, we independently scored insulitis levels and changes in Myt3 signal in pancreas sections from 12-week old female NOD mice (Figure 7B, C). From this analysis it was evident that as insulitis progresses there is a concomitant decrease in Myt3 expression. Together, these data indicate that cytokines that cause β-cell dysfunction and apoptosis negatively regulate *Myt3* expression and that this may be relevant to the progression of diabetes in NOD mice.

### *Myt3* Suppression Reduces Insulin Content in β-cells

**Figure 8:**
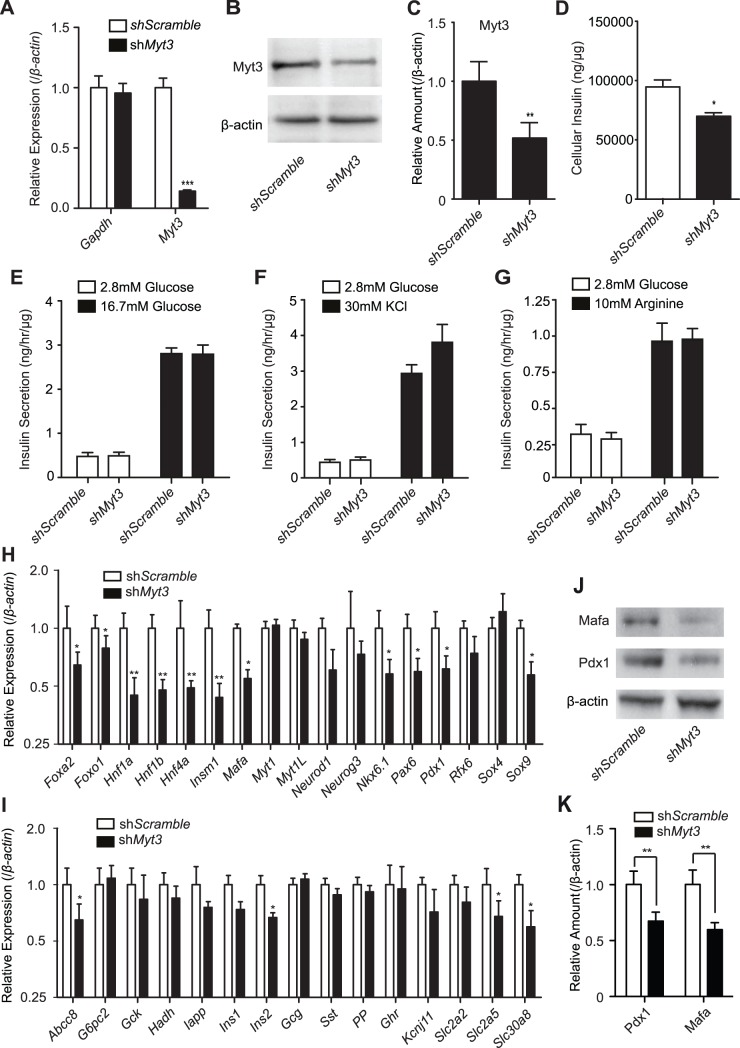
*Myt3* regulates insulin content and gene expression in β-cells. Islets were transduced with adenoviruses expressing shRNA’s targeting *Myt3* or a scrambled sequence. **A)** *Myt3* expression was determined relative to *β-actin. Gapdh* was used as a control for off target effects of the virus. **B)** Western blot analysis of Myt3 and β-actin protein levels in islets. **C)** Results of the densitometry of triplicate western blot analyses from **B** relative to β-actin. *Ex vivo* islets were transduced as above and **D)** cellular insulin content and insulin secretion induced with **E)** 16.7 mM Glucose, **F)** 30 mM KCl or **G)** 10 mM Arginine were determined 48 hrs later by Insulin ELISA. qPCR was used to determine the relative expression of **H)** transcription factors and cofactors and **I)** Genes involved in β-cell function/physiology as compared to *β-actin*. **J)** Western blot analysis of Mafa, Pdx1, and β-actin protein levels. **K)** Results of the densitometry of triplicate western blot analyses from **J** relative to β-actin. * indicates a statistically significant difference at p≤0.05, ** at p≤0.01, *** at p≤0.001 based on students t-test.

To determine whether *Myt3* plays a role in regulating glucose-stimulated insulin secretion we generated three independent adenoviruses expressing shRNA sequences targeting *Myt3* (sh*Myt3*) (see methods and materials) or a scramble sequence (sh*Scramble*). qPCR analysis of FACS-sorted islets indicated that clone TRCN0000042479 resulted in the highest level of *Myt3* suppression (data not shown) and this clone was used in all subsequent experiments. Our analysis also showed the sh*Myt3* virus had no effect on *Gapdh* expression, but reduced *Myt3* levels by approximately 5-fold (p≤0.001, n = 10) as compared with islets treated with the sh*Scramble* virus (Figure 8A). Treatment of whole islets with the sh*Myt3* virus also significantly reduced Myt3 protein level by 2-fold (p≤0.01, n = 3) (Figure 8B, C). *Myt3* suppression in islets modestly, but significantly (1.4-fold, p≤0.05, n = 3) reduced cellular insulin levels (Figure 8D), but had no effect on their ability to secrete insulin following stimulation with glucose, KCl or arginine (Figure 8E–G).

To determine how suppression of *Myt3* reduces cellular insulin levels we assessed the effect of *Myt3* suppression on the expression of selected transcriptional regulators important in pancreas development or function, or genes with well established roles in β-cell function. *Myt3* suppression in *ex vivo* islets had a significant effect on several transcription factors and cofactors known to regulate β-cell function, including *Hnf1*α, *Hnf1*β, *Hnf4*α, *Insm1*, *Sox9*, *Pdx1*, and *Mafa*, which were all reduced by at least 1.6-fold (Figure 8H). Of the genes involved in β-cell function, *Myt3* suppression reduced *Abcc8* and *Slc30a8* the most, by 1.54-fold and 1.67-fold respectively (Figure 8I). *Myt3* suppression also impaired *Ins1* and *Ins2* expression, while the expression levels of the other islet hormones were unaltered (Figure 8I). Treating MIN6 cells with siRNA’s targeting *Myt3* produced similar results for selected genes, in particular for *Pdx1* and *Mafa* (data not shown). Given this, and as Pdx1 and Mafa have well-established roles in β-cell function [10], [12], we attempted to validate their repression at the protein level. Western blot analysis of islets transduced with adenovirus expressing sh*Myt3* reduced Mafa levels by 1.67-fold (p<0.001, n = 3) and Pdx1 levels by 1.48-fold (p<0.001, n = 3) (Figure 8J, K), consistent with our qPCR data. These results suggest that *Myt3* affects cellular insulin content via the regulation of several genes including *Ins1, Ins2, Pdx1* and *Mafa.*

### *Myt3* Regulates β-cell Survival

**Figure 9:**
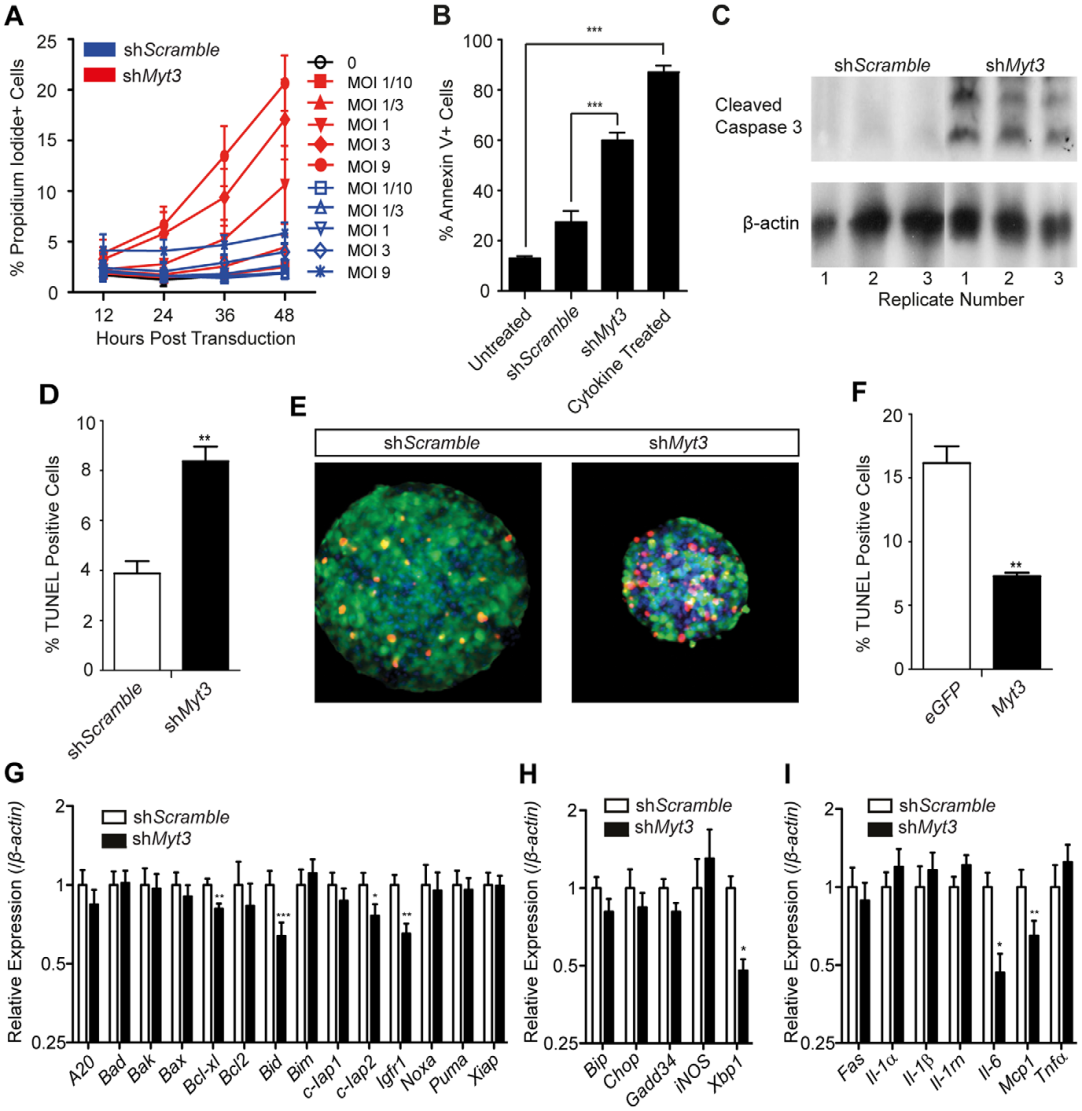
*Myt3* is critical for β-cell survival. **A)** The percent of MIN6 cells that were PI positive at the indicated time points after being transduced with varying amounts of adenoviruses expressing shRNA’s targeting *Myt3* or a scrambled sequence. **B)** Quantification of Annexin-V APC positive cells in virus treated MIN6 cells. Untransfected cells were used as a negative control while cytokine treated cells acted as a positive control. **C)** Western blot analysis of cleaved Caspase 3 and β-actin levels in transduced MIN6 cells. Numbers indicate separate biological replicates. **D)** Quantification of TUNEL positive cells in dispersed islets treated with *shScramble* or *shMyt3* viruses. **E)** Representative images of TUNEL staining (red) of transduced islets. Transduced cells are stained in green and nuclei are labelled with Hoechst (blue). **F)** Quantification of TUNEL positive cells in dispersed islets treated with *eGFP* or *Myt3* over-expression viruses. **G)** Expression of pro- and anti-apoptotic genes relative to β-Actin. * indicates a statistically significant difference at p≤0.05, ** at p≤0.01, *** at p≤0.001 based on students t-test.

Exposure of islets to cytokines both *in vitro* and *in vivo* suppresses *Myt3* expression suggesting a potential role for *Myt3* in β-cell survival. To test this hypothesis we transduced MIN6 cells with our adenoviruses expressing shRNA’s targeting *Myt3* or a scramble sequence and incubated the cells with propidium iodide (PI). Increasing sh*Myt3* virus concentration significantly increased β-cell death over time (p≤0.0001, n = 4) (Figure 9A). Similarly, *Myt3* suppression increased Annexin-V positive cells by 2-fold (p≤0.001, n = 3) (Figure 9B), and the level of cleaved caspase 3 (Figure 9C). To validate these results we performed TUNEL analysis on dispersed islets treated with either the *shScramble* or *shMyt3* virus. Our data show that apoptosis was increased by approximately 2-fold (p≤0.01, n = 4) (Figure 9D), similar to our results in MIN6 cells. This was also confirmed in whole islets (Figure 9E). As cytokine exposure results in reduced *Myt3* expression, and adenoviral mediated suppression of *Myt3* increases apoptosis, we examined the ability of *Myt3* over-expression to protect islets from cytokine mediated cell death. Dispersed islets treated with an adenovirus over-expressing *Myt3* had a greater than 2-fold (p≤0.01, n = 4) decrease in cytokine induced apoptosis, as compared to islets treated with a control adenovirus expressing eGFP, as revealed by TUNEL staining (Figure 9F).

To determine how *Myt3* regulates apoptosis in β-cells we examined the expression of a number of different anti-apoptotic and pro-apoptotic genes in sh*Myt3* and sh*Scramble* treated islets. Our data demonstrate that *Myt3* suppression leads to a 1.25-fold (p≤0.01, n = 3) reduction in *Bcl-xl,* a 1.54-fold (p≤0.01, n = 3) reduction in *Igfr1* and a 1.4-fold (p≤0.05, n = 3) reduction in *c-Iap2* (Figure 9G). To determine whether endoplasmic reticulum (ER) stress played a role in these changes we assessed the expression of genes characteristic of ER stress [49], [50]. We found that *Bip, CHOP, Gadd34* and *iNOS* were unchanged, however, *Xbp1* was reduced 2-fold (p≤0.05, n = 3) (Figure 9H). Finally, as *Myt3* plays a role in pro-inflammatory gene expression in fibroblasts, we further assessed the expression of selected β-cell expressed cytokines. *Myt3* suppression caused a 2-fold (p≤0.05, n = 3) reduction in *Il-6* expression but had no effect on the expression levels of *Il-1α, Il-1β, Il-1rn* or *Tnfα* (Figure 9I). Together, these results indicate that *Myt3* plays a significant role in regulating β-cell survival and pro-inflammatory gene expression.

## Discussion

We anticipated that the identification of transcription factors specifically expressed in developing endocrine cells, or in adult pancreatic islets, would provide insight into the transcriptional networks that regulate β-cell genesis and function [2]. In trying to find such factors we identified *Myt3*. *Myt3* has a high degree of similarity to other MYT family members, particularly *Myt1*, with both genes encoding proteins with conserved zinc-finger, and MYT family domains [16]. Furthermore, both transcription factors recognise similar synthetic oligonucleotides, with Myt1 recognizing the consensus sequence RRRAGTT, and Myt3 recognizing the related AAASTTT consensus sequence, suggesting some degree of functional redundancy [16], [31]. Previous reports indicated that the MYT family of transcription factors is highly expressed in neural tissue [51], [52], but that only *Myt1* is expressed in developing pancreas cells [23]. Our data agree with these reports and indicate that *Myt1l* and *Myt3* have little or no expression early in pancreas development [25]; however our SAGE, qPCR, and IHC data indicate that *Myt3* is relatively abundant in mature pancreatic islets. In fact, *Myt3* is greater than 10-fold more highly expressed in islets than either *Myt1* or *Myt1l*. Furthermore, *Myt3* is expressed in human islets, albeit at a lower level than in mouse islets, suggesting that *Myt3* is important not only for islet function in rodents, but also in humans.

In the pancreas, endocrine progenitors are specified by the expression of *Ngn3* during the secondary transition (∼E13.5) [34], [53]. During this time frame *Ngn3* expressing cells differentiate and expand. Subsequently, from ∼E16.5 until several days after birth, these cells coalesce into islet structures and increase their expression of key maturation factors such as *Neurod1* and *Mafa* that drive their maturation into fully functional endocrine cells [53], [54]. Myt3 protein first appears in endocrine cells at ∼E18.5 during the period of islet maturation and is maintained in mature α-, β-, δ-, and PP-cell types. The expression of Myt3 from E18.5 onwards suggests that it may play an important role in the regulation of this maturation step and in the maintenance of mature β-cell function.

The tightly controlled spatiotemporal expression of *Myt3* suggests precise, tissue specific transcriptional regulation. We show that the *Myt3* promoter is bound and directly regulated by Foxa2, Pdx1 and Neurod1. Foxa2 is a critical initiator of *Pdx1* expression [55] and loss of either transcription factor leads to impaired pancreas development and perinatal lethality [55], [56]. Foxa2 and Pdx1 are both expressed in mature β-cells where they function to regulate insulin vesicle docking to the plasma membrane and insulin biosynthesis respectively [11], [57]. Meanwhile, Neurod1 is essential for specification and differentiation of endocrine cell types and also functions in mature β-cells to regulate insulin biosynthesis and secretion [13], [57], [58]. The regulation of the *Myt3* promoter by Foxa2, Pdx1 and Neurod1 suggests that it may play an important role in mediating the downstream effects of these transcription factors.

Genes that are maintained in the adult islet by Neurod1 are often induced by Ngn3 during development, as both bind similar E-box elements [35]. In concordance, Neurod1 and Ngn3 induce similar sets of genes when over-expressed in mPAC cells [36]. The importance of the identified E-box element in the *Myt3* promoter in initiating and maintaining its expression is exemplified by the fact that not only does Ngn3 induce *Myt3* expression in mPAC cells but Neurod1 over-expression also has the most significant affect on *Myt3* promoter activity relative to Foxa2 and Pdx1. In addition, ectopic expression of *Ngn3* induces a more open and active chromatin state around the *Myt3* promoter, through an increase in the enrichment of the activating H3K4me1 and H3K27ac marks, with a concomitant decrease in repressive H3K27me3 enrichment levels. These data suggest that Ngn3 induced changes to the histone modification state around the *Myt3* promoter may allow it to become activated by other factors, and that once activated its expression is maintained in mature islets, at least in part, by Neurod1 and Pdx1.

Pancreatic islets respond to elevated glucose levels following feeding, not only by secreting insulin, but also by increasing insulin, and other, gene expression [59]. These functional responses are mediated, in part, through the glucose-induced translocation of Pdx1 and Neurod1 to the nucleus where they can affect gene expression changes [33], [60]. As we determined that both of these factors are direct regulators of *Myt3* expression we evaluated the role of glucose in the regulation of *Myt3* expression. In fact, increasing glucose concentrations resulted in increased *Myt3* expression. Exposure of islets to elevated glucose levels increases *Insulin* expression within one hour of exposure [33], [60], [61]; however, *Myt3* expression was only up-regulated after 6 hrs post transfer to 16.7 mM glucose. To determine whether this delay was due to a need for synthesis of regulatory proteins we inhibited protein synthesis with cycloheximide. Surprisingly, treatment with cycloheximide increased basal *Myt3* expression likely due to removal of inhibitory factors with high protein turnover rates. Cycloheximide, however, did not affect the ability of 16.7 mM glucose to induce *Myt3* expression suggesting a mechanism other than new protein synthesis for the delay in up-regulation of *Myt3* expression. It is possible that the delay is the result of a need to recruit additional transcription factors, or to a more restrictive epigenetic landscape that needs to be altered to facilitate increased gene expression. Regardless, these data indicate that *Myt3* expression is glucose responsive.

In addition to being glucose responsive, both *Pdx1* and *Neurod1* are also repressed by exposure to the pro-inflammatory cytokines Il-1β, TNFα and IFNγ. [62], [63]; we therefore further examined the effect of these cytokines on *Myt3* expression. Exposure of islets to Il-1β, TNFα and IFNγ *in vitro* resulted in a significant reduction in *Myt3* expression. Furthermore, in a mouse model of T1D, immune infiltration into the islet results in a concomitant reduction in *Myt3* expression likely due to exposure of the islets to pro-inflammatory cytokine secretion from the infiltrating immune cells confirming the *in vivo* relevance of our cytokine results.

Prolonged exposure to cytokines, which occurs in type 1 (T1D) and type 2 (T2D) diabetes, results in β-cell dysfunction and apoptosis [64], [65]. We initially wondered whether cytokine induced β-cell dysfunction may be mediated through *Myt3* suppression. To determine this we assessed whether shRNA mediated *Myt3* suppression could impair islet function. Although, *Myt3* suppression did not cause any change in glucose-, KCl-, or arginine-stimulated insulin secretion, *Myt3* suppression did reduce intra-cellular insulin content. To begin to assess the mechanism underlying the reduced insulin content we interrogated the gene expression of several factors with known roles in regulating β-cell function and insulin gene expression. We show that *Myt3* regulates many of these factors, including *Pdx1* and *Mafa,* which function synergistically to regulate insulin expression levels [57]. In agreement, *Myt3* suppression also reduced *Ins1* and *Ins2* expression levels. Also, consistent with the lack of impaired insulin secretion, genes involved in insulin secretion were mostly unaltered. Thus, we suggest that while the level of *Myt3* suppression we are able to achieve in whole islets is unable to induce defects in glucose-, KCl-, and arginine-stimulated insulin secretion, it is sufficient to alter cellular insulin levels due, at least in part, to reduced Pdx1 and Mafa levels.

We next assessed whether cytokine induced β-cell apoptosis might be mediated through *Myt3* suppression. In fact, our data clearly show that Myt3 suppression leads to increased apoptosis in islets and MIN6 cells, suggesting that Il-1β, TNFα and IFNγ induced *Myt3* repression may be a significant factor in cytokine induced β-cell apoptosis. We further demonstrate that adenoviral mediated *Myt3* over-expression largely prevents cytokine-induced apoptosis in islets. In agreement with *Myt3* having a pro-survival role in β-cells, suppression of *Myt3* resulted in a significant reduction in the expression of *Bcl-xl,* which alters the localisation of the pro-apoptotic Bax from the mitochondrial membrane to the cytoplasm thus preventing cytochrome c release and subsequently apoptosis [66], and *c-Iap2* that regulates cell survival via inhibition of effector caspase activity [67]. Also, *Il-6* and *Igfr1* expression were significantly reduced by *Myt3* suppression. *Il-6* induces α-cells to secrete the incretin hormone GLP-1 [68]. GLP-1 stimulates β-cell *Igfr1* expression, which regulates β-cell survival via Akt signalling [69], [70]. This suggests that *Myt3* may indirectly affect β-cell survival by reducing levels of Il-6 induced GLP-1 secretion from α-cells, thereby reducing *Igfr1* and thus increasing β-cell apoptosis; although, further work is required to validate this model. Further, *Il-6* has been shown to protect islets from pro-inflammatory cytokine exposure both *in vitro* and *in vivo* [71]. In addition, *Pdx1* and *Mafa* also play pro-survival roles in β-cells [72], [73]. In fact, similar to our findings, increased β-cell apoptosis in *Pdx1* heterozygous mice is due to reduced expression of the pro-survival factors *Bcl2* and *Bcl-xl* [72]. In further agreement, insulin secretion is similarly unimpaired in these mice [72]. Thus, the *Myt3* suppression induced reductions in *Pdx1* levels that we note, and the phenotype we see, are consistent with the phenotype of *Pdx1* heterozygous mice that have similar levels of *Pdx1* in their islets. Together, these data clearly demonstrate that changes in *Myt3* expression levels are sufficient to alter apoptosis in islets, likely through the regulation of pro-survival genes such as *Pdx1*, *Il-6*, *Bcl-xl*, *c-Iap2*, and *Igfr1*.

In summary, we have identified *Myt3* as the predominant MYT family member in mature islets, and show that it is present in all major endocrine cell types. We show that *Myt3* expression is regulated by the transcription factors Foxa2, Pdx1 and Neurod1 and that its expression is responsive to both glucose and cytokines. We demonstrate that *Myt3* suppression reduces cellular insulin levels, and significantly increases the rate of β-cell apoptosis. Importantly, over-expression of *Myt3* is able to protect cells from cytokine-induced apoptosis. These data are an important step in clarifying the regulatory networks responsible for β-cell function and survival, and suggest that *Myt3* may be an interesting therapeutic target for improving β-cell survival in diabetic patients and islet graft recipients.
